# Supplementary material for: Characterization of Terpenoids in Aromatic Plants Using Raman Spectroscopy and Gas Chromatography–Mass Spectrometry (GC–MS)
Source: Int J Mol Sci. 2025 Nov 21;26(23):11254. doi: 10.3390/ijms262311254 (PMC12692706; doi:10.3390/ijms262311254)
Supplement: Supplementary file 1 [file ijms-26-11254-s001.zip › ijms-3971039-supplementary.pdf]

a) **b)**

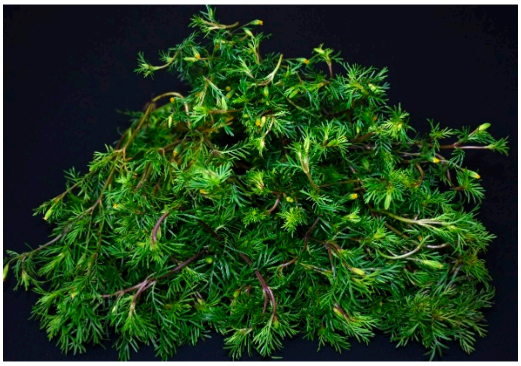

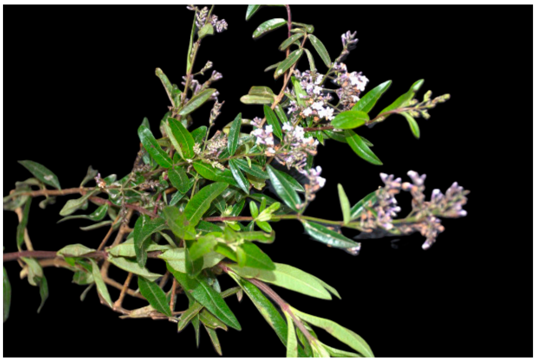

c) **d)**

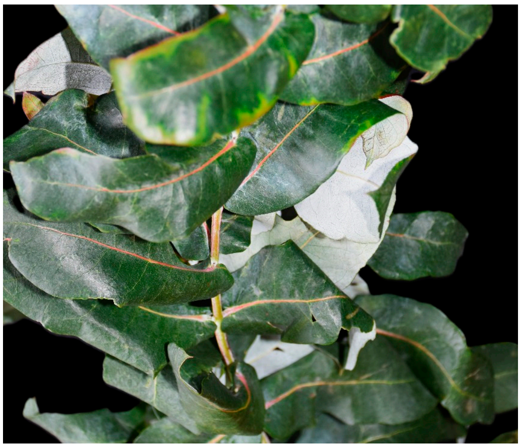

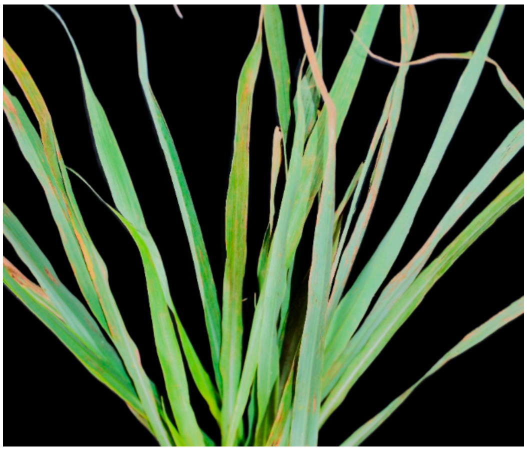

e) **f)**

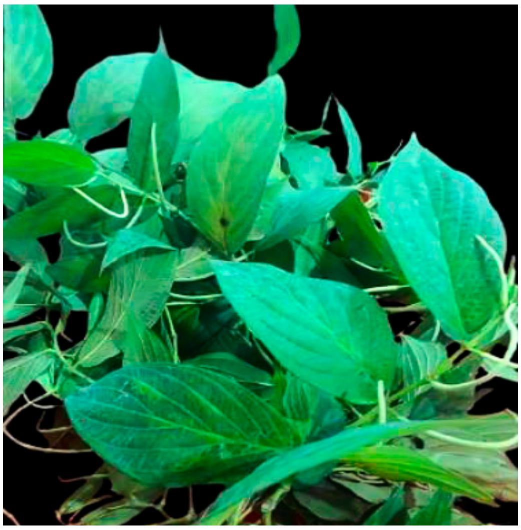

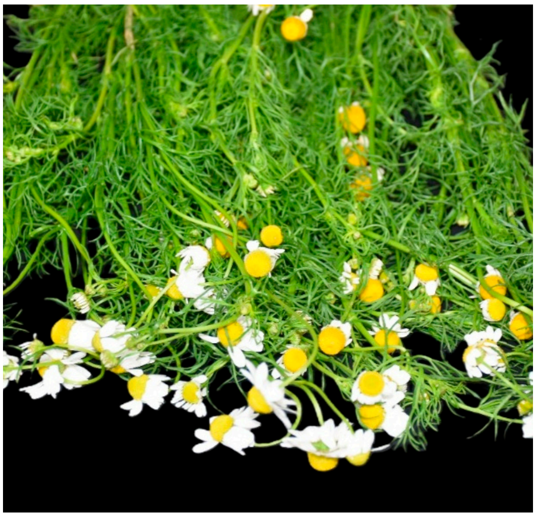

g) **h)**

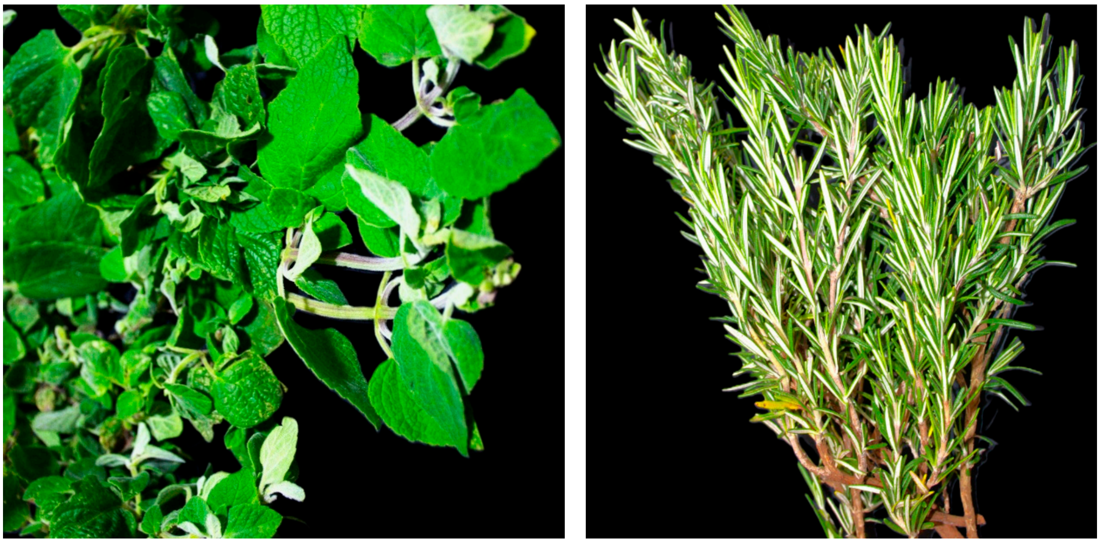

Figure S1. Representative photographs of the plant species studied: *(a) Tagetes filifolia (b) Aloysia citrodora, (c) Eucalyptus globulus, (d) Cymbopogon citratus, (e) Piper aduncum, (f) Matricaria chamomilla, (g) Minthostachys mollis, (h) Rosmarinus officinalis.*

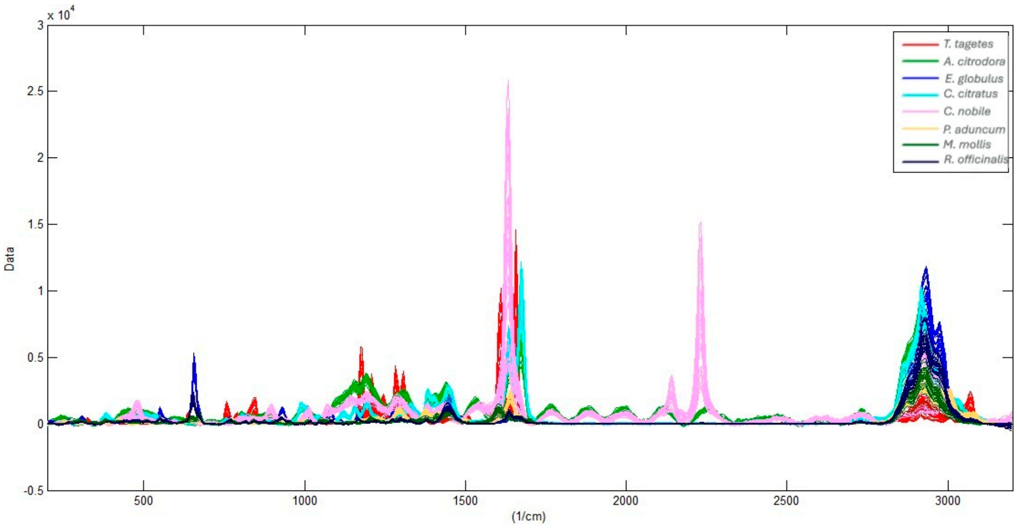

Figure S2. Averaged Raman spectra of *Tagetes filifolia, Aloysia citrodora, Eucalyptus globulus, Cymbopogon citratus, Chamaemelum nobile, Piper aduncum, Minthostachys mollis,* and *Rosmarinus officinalis* in the 200–3100 cm^{-1} region

Table S1. Chemical composition profile of essential oils analyzed by GC-MS

Chemical families	Compounds	*T. filifolia*	*A. citrodora*	*E. globulus*	*C. citratus*	*P.aduncum*	*M. mollis*	*R. officinalis*
**Alcohol**	**1-Octen-3-Ol**	0.00	1.54	0.00	0.00	0.00	0.00	1.63
	**9-Dodecyn-1-ol**	0.00	0.00	0.00	0.00	0.00	0.46	0.00
	**3-Nonanol**	0.00	0.00	0.00	0.00	0.00	0.12	0.00
	**3-Octanol**	0.00	0.16	0.00	0.00	0.00	2.50	0.00
**Aldehyde**	**Acetaldehyde, (3,3-dimethylcyclohexylidene)-, (Z)-**	0.00	0.00	0.00	0.00	0.16	0.00	0.00
	**Butanal, 3-methyl-**	0.00	0.00	0.24	0.00	0.00	0.00	0.00
	**Nonanal**	0.00	0.10	0.00	0.00	0.00	0.00	0.00
	**Pentanal, 5-(methylenecyclopropyl)-**	0.00	0.00	0.00	0.00	0.00	0.50	0.00
	**p-Anisaldehyde**	10.22	0.00	0.00	0.00	0.00	0.00	0.00
**Ester**	**3-Hexen-1-ol, acetate, (E)-**	0.00	0.00	0.25	0.00	0.00	0.00	0.00
	**2-Fluorobenzoic acid, 4-nitrophenyl ester**	0.00	0.25	0.00	0.00	0.00	0.00	0.00
	**Isoamyl acetate**	0.00	0.00	0.26	0.00	0.00	0.00	0.00
	**Texanol Isobutyrate**	0.00	0.00	0.17	0.00	0.00	0.00	0.00
	**Isovalerate**	0.00	0.00	0.21	0.00	0.00	0.00	0.00
	**Phenethyl acetate**	0.00	0.00	1.28	0.00	0.00	0.00	0.00
	**Phenethyl isovalerate**	0.00	0.00	0.28	0.00	0.00	0.00	0.00
	**3-Octanol, acetate**	0.00	0.00	0.00	0.00	0.00	1.75	0.00
**Hydrocarbon**	**Pentadecane**	0.00	0.00	0.00	0.00	0.84	0.00	0.00
	**1-Undecene**	1.03	0.00	0.00	0.00	0.00	0.00	0.00
	**Toluene**	0.73	0.00	0.00	0.00	0.00	0.00	0.00
	**(3E,5Z)-1,3,5-Undecatriene**	0.00	0.00	0.00	0.00	0.26	0.00	0.00
	**Apiol**	10.63	0.17	0.00	0.15	0.42	0.91	0.22
**Ketone**	**2,4,6-Tris(1,1-dimethylethyl)-4-methylcyclohexa-2,5-dien-1-one**	1.60	0.19	0.00	0.00	0.00	0.00	0.00
	**2-Oxabicyclo[2.2.2]octan-6-ol, 1,3,3-trimethyl-, acetate**	0.00	1.02	0.50	0.00	0.00	0.00	0.00
	**2-Cyclopenten-1-one, 2,3-dimethyl-**	0.00	0.00	0.00	0.00	0.00	0.40	0.00
	**2-Butil-2-ciclopenten-1-ona**	0.00	0.00	0.00	0.00	0.13	0.00	0.00
	**Neophytadiene**	2.15	0.00	0.00	0.00	0.00	0.00	0.00
	**2-Tridecanone**	0.00	0.00	0.00	1.02	0.00	0.00	0.00
	**2-Undecanone**	0.00	0.00	0.00	1.79	0.00	0.00	0.00
	**Piperitone**	0.00	0.00	0.00	0.00	0.00	3.25	0.00
	**Piperitenone**	0.00	0.36	0.00	0.00	0.00	2.68	0.00
	**5,9-Undecadien-2-one, 6,10-dimethyl-, (E)-**	0.00	0.00	0.00	0.00	0.00	0.13	0.00
	**4-Propanoylanisole**	0.38	0.00	0.00	0.00	0.00	0.00	0.00
	**4-Methoxyphenylacetonea**	2.61	0.00	0.00	0.00	0.00	0.00	0.00
	**3-Octanone**	0.00	0.00	0.00	0.00	0.00	0.25	0.65

**Others**	**Others compounds**	0.00	1.09	1.07	2.42	1.53	1.04	1.65
**Phenolic**	**Butylated hydroxytoluene**	0.00	0.00	0.83	0.23	0.00	0.00	0.36
**Phenylpropanoid**	**1-(4-Methoxyphenyl)propane-1,2-diol**	0.38	0.00	0.00	0.00	0.00	0.00	0.00
	**Myristicin**	0.97	0.00	0.00	0.00	2.42	0.00	0.00
	**3,5-Dimethoxycinnamic acid**	0.00	0.00	0.00	0.00	0.47	0.00	0.00
	**Chavicol**	0.68	0.00	0.00	0.00	0.00	0.00	0.00
	**Anethole**	1.31	0.16	0.00	0.15	0.00	1.16	0.00
	**Benzene, 1,2-dimethoxy-4-(1-propenyl)-, (E)-**	1.06	0.00	0.00	0.00	0.00	0.00	0.00
	**Benzene, 1,4-dimethoxy-2-methyl-5-isopropyl-**	3.61	0.00	0.00	0.00	0.00	0.00	0.00
	**Eugenol**	0.00	0.41	0.00	0.00	0.00	0.00	0.00
	**Methyleugenol**	3.61	0.00	0.00	0.00	0.00	0.00	0.00
	**Safrole**	0.00	0.00	0.00	0.00	11.55	0.00	0.00
**Terpenoid**	**(3R,6R)-3-Hydroperoxy-3-methyl-6-(prop-1-en-2-yl)cyclohex-1-ene**	0.00	0.00	0.13	0.00	0.00	0.00	0.00
	**cis,cis-Nepetalactone**	0.00	0.00	0.00	0.00	0.00	30.16	0.00
	**gamma-Terpinene**	0.00	0.00	0.63	0.00	3.06	0.00	5.78
	**alpha Terpinene**	0.00	0.00	0.13	0.00	1.35	0.00	2.51
	**Ocimenol, (E)-**	0.00	0.00	0.00	0.12	0.00	0.00	0.00
	**4-Menthen-8-ol**	0.00	0.00	0.00	0.00	0.00	0.14	0.00
	**Rosefuran epoxide**	0.00	0.74	0.00	0.16	0.00	0.00	0.00
	**Citral / GERANIAL**	2.35	0.00	0.40	0.00	0.00	0.00	0.00
	**Nerol**	0.00	2.46	0.00	0.84	0.10	0.00	0.00
	**Geranic acid**	0.00	0.00	0.00	1.51	0.00	0.00	0.00
	**Neric acid**	0.00	0.00	0.00	0.56	0.00	0.00	0.00
	**Rose oxide**	0.00	0.00	0.00	0.12	0.00	0.00	0.00
	**E-2-Caren-4-ol**	0.00	0.00	0.00	0.00	0.00	0.00	0.13
	**2-Cyclohexen-1-ol, 2-methyl-5-(1-methylethenyl)-, cis-**	0.00	0.00	0.19	0.00	0.00	0.00	0.45
	**2-Ciclohexen-1-ol, 1-metil-4-(1-metiletil)-, trans-**	0.00	0.00	0.00	0.00	0.12	0.00	0.00
	**Carvyl acetate**	0.00	0.00	0.00	0.00	0.00	0.15	0.00
	**2-Hydroxypiperitone**	0.00	0.00	0.00	0.00	0.00	0.69	0.00
	**(+)-Isopiperitenone**	0.00	0.00	0.00	0.00	0.00	0.40	0.00
	**Cinerolone**	0.00	0.00	0.00	0.00	0.00	0.55	0.00
	**alpha-Pinene oxide**	0.00	0.00	0.17	0.00	0.00	0.00	0.00
	**3-Carene**	0.00	0.00	0.00	0.00	0.00	0.12	0.00
	**3-Cyclohexene-1-methanol, 5-hydroxy-.alpha.,.alpha.,4-trimethyl-**	0.00	0.00	0.00	0.14	0.00	0.00	0.00
	**3-Cyclohexen-1-ol, 4-methyl-1-(1-methylethyl)-, (R)-**	0.00	0.00	0.00	0.00	0.00	0.00	2.51
	**3-Cyclopentene-1-ethanol, 2,2,4-trimethyl-**	0.00	0.00	0.00	0.00	0.00	0.00	4.33
	**3-Cyclohexen-1-one, 2-isopropyl-5-methyl-**	0.00	0.00	0.00	0.00	4.25	0.00	0.00

4,7,7-Trimethylbicyclo[4.1.0]hept-3-en-2-one	0.00	0.17	0.00	0.00	0.00	0.00	0.00
5-Caranol, trans,trans-(+)-	0.00	0.00	0.00	0.00	0.00	0.00	3.74
5-Hepten-1-ol, 2,6-dimethyl-	0.00	0.66	0.00	0.00	0.00	0.00	0.00
5-Hepten-2-one, 6-methyl-	0.00	1.27	0.00	2.21	0.00	0.00	0.00
5-Heptenal, 2,6-dimethyl-	0.00	0.48	0.00	0.00	0.00	0.00	0.00
Sabinene hydrate	0.00	0.22	0.00	0.00	0.00	0.00	0.77
6-Hydroxycarvotanacetone	0.00	0.00	0.00	0.00	0.00	0.94	0.00
Citronellol acetate	0.00	0.60	0.00	0.00	0.00	0.00	0.00
6-Octen-1-ol, 3,7-dimethyl-, (R)-	0.00	0.00	0.00	1.42	0.00	0.00	0.00
6-Octenal, 3,7-dimethyl-, (R)-	0.00	0.00	0.00	0.44	0.00	0.00	0.00
6-Octenal, 7-methyl-3-methylene-	0.00	0.00	0.00	0.67	0.00	0.00	0.00
Citronellyl propionate	0.00	0.16	0.00	0.00	0.00	0.00	0.00
Citronellic acid	0.00	0.30	0.00	0.00	0.00	0.00	0.00
Methyl citronellate	0.00	0.98	0.00	0.00	0.00	0.00	0.00
Limonene dioxide	0.00	0.00	0.00	0.00	0.00	0.16	0.00
8,9-Dehydrothymol	0.00	0.00	0.00	0.00	0.00	0.41	0.00
beta-Ciclocitral	0.00	0.00	0.00	30.42	0.00	0.00	0.00
Myrcene	0.58	0.00	8.49	0.00	0.00	0.39	0.00
Bornylene	0.00	0.00	0.00	0.00	0.00	0.00	0.18
Bicyclo[2.2.1]heptane, 2,2-dimethyl-5-methylene-	0.00	0.00	0.00	0.00	0.00	0.00	9.97
alpha-Fenchene	0.00	0.00	0.13	0.00	0.00	0.00	0.18
Alpha-Thujene	0.00	0.00	1.07	0.00	0.22	0.00	1.20
Sabinene	0.00	0.00	0.00	0.00	0.00	0.86	0.49
Verbenol, (S)-cis-	0.00	0.00	0.00	0.00	0.00	0.00	0.12
Bicyclo[3.1.1]hept-3-en-2-one, 4,6,6-trimethyl-, (1S)-	0.00	0.00	0.00	0.00	0.00	0.00	2.18
2-Pinanol	0.00	0.00	0.00	0.00	0.00	0.00	0.11
Geranyl butyrate	0.00	0.24	0.00	0.00	0.00	0.00	0.00
Carveol	0.00	0.00	0.00	0.17	0.00	0.00	0.00
Camphene	0.00	0.00	0.31	0.00	0.00	0.00	0.00
beta-Caryophyllene	0.00	6.00	0.00	0.22	11.00	0.00	18.26
Caryophyllene oxide	0.00	0.92	0.34	0.28	0.53	0.00	1.04
Copaene	0.00	0.63	0.00	0.00	3.14	2.94	0.00
Citronellol	0.00	10.92	0.00	0.00	0.00	0.00	0.00
cis-Verbenol	0.00	0.00	0.74	0.00	0.00	0.00	0.00
Cyclohexene, 4-ethenyl-4-methyl-3-(1-methylethenyl)-1-(1-methylethyl)-, (3R-trans)-	0.00	0.00	0.00	0.00	0.20	0.00	0.00
Cyclohexanol, 5-methyl-2-(1-methylethenyl)-	0.00	0.23	0.00	0.00	0.00	0.00	0.00
Cyclohexanol, 5-methyl-2-(1-methylethyl)-, (1.alpha.,2.alpha.,5.alpha.)-	0.00	0.00	0.00	0.00	0.00	6.82	0.00

**Cyclohexanol, 5-methyl-2-(1-methylethyl)-, acetate, (1.alpha.,2.alpha.,5.beta.)-**	0.00	0.00	0.00	0.00	0.00	1.11	0.00
**Cyclohexanone, 2-methyl-5-(1-methylethenyl)-, trans-**	0.00	0.00	0.00	0.00	0.00	0.25	0.00
**Isopulegone**	0.00	0.00	0.00	0.00	0.00	0.49	0.00
**Cyclohexanone, 5-methyl-2-(1-methylethyl)-, cis-**	0.00	0.00	0.00	0.00	0.00	3.16	0.00
**Terpinolene**	0.00	0.00	0.00	0.00	0.00	0.15	3.07
**Dihydrocarvenyl acetate(equatorial)**	0.00	0.26	0.00	0.00	0.00	0.00	0.00
**Isocitral**	0.00	0.56	0.00	4.13	0.00	0.00	0.00
**Isogeraniol**	0.00	0.18	0.00	0.00	0.00	0.00	0.00
**Isoneral**	0.00	0.93	0.00	2.96	0.00	0.00	0.00
**L-.alpha.-Terpineol**	0.00	0.00	0.00	0.00	2.97	0.00	5.24
**Lavandulol**	0.00	0.10	0.00	0.00	0.00	0.00	0.00
**Linalool**	1.61	0.59	0.90	2.33	0.33	9.63	2.91
**Modephene**	2.14	0.00	0.00	0.00	0.00	0.00	0.00
**Neral**	1.42	0.00	0.21	0.00	0.15	0.00	0.00
**Pinocarvone**	0.00	0.00	0.00	0.00	0.00	0.00	0.61
**Pulegone**	0.00	0.28	0.00	0.17	0.00	0.00	0.00
**Terpinen-4-ol**	0.00	0.15	3.54	0.00	2.03	0.00	0.00
**Terpinolene**	0.00	0.00	1.08	0.00	0.00	0.00	0.00
**trans-.beta.-Ocimene**	0.00	0.14	3.15	1.01	9.01	0.00	0.00
**trans-Ascaridol glycol**	0.00	0.00	0.13	0.00	0.00	0.00	0.00
**trans-verbenol**	0.00	0.00	0.25	0.00	0.00	0.00	0.43
**Thymol**	0.00	0.00	0.00	0.00	0.00	2.04	0.00
**Thymoquinone**	0.00	0.00	0.00	0.00	0.00	0.20	0.00
**Cyclofenchene**	0.00	0.00	0.00	0.00	4.17	0.67	0.53
**alpha-Campholenal**	0.00	0.00	0.21	0.00	0.00	0.00	0.48
**alpha-Phellandrene**	0.00	0.00	0.54	0.00	2.92	0.00	0.80
**beta-Phellandrene**	0.00	0.50	0.00	0.00	1.31	0.10	0.00
**alpha-Terpinyl acetate**	0.00	0.00	18.26	0.00	0.00	0.00	0.00
**alpha-Terpineol**	0.00	0.86	14.25	0.00	0.00	0.68	0.00
**alpha-Pinene**	1.27	0.12	0.00	0.00	0.00	0.00	0.00
**Mirtenol**	0.00	0.00	0.11	0.00	0.00	0.00	0.00
**(S)-cis-Verbenol**	0.00	0.00	0.14	0.00	0.00	0.00	0.00
**Pinocarveol**	0.00	0.00	0.40	0.00	0.00	0.00	0.00
**Mirtanal**	0.00	0.16	0.00	0.15	0.00	0.00	0.00
**Bornyl acetate**	0.00	0.00	0.00	0.00	0.00	0.00	4.99
**D-Limonene**	2.81	6.31	0.00	0.28	4.59	6.87	8.59
**Eucalyptol**	0.35	1.42	0.00	0.00	0.00	0.29	0.00
**Epoxy-linalooloxide**	0.00	0.00	0.00	0.38	0.00	0.00	0.00
**Fenchol**	0.00	0.00	0.24	0.00	0.00	0.00	0.00
**Perillene**	0.00	0.00	0.00	0.35	0.00	0.00	0.00
**Geraniol**	0.39	3.17	3.13	6.06	0.00	0.00	0.00
**Geranyl acetate**	0.00	0.00	1.42	0.59	0.00	0.00	0.00
**Geranyl propionate**	0.00	0.85	0.00	0.00	0.00	0.00	0.00

**2,3-Epoxigerianial**	0.00	0.00	0.00	0.11	0.00	0.00	0.00
**p-Cymene**	0.00	0.00	6.63	0.00	0.00	0.00	2.77
**Neomenthoglycol**	0.00	0.11	0.00	0.00	0.00	0.00	0.00
**Cyclohexanemethanol, .alpha.,.alpha.-dimethyl-4-methylene-**	0.00	0.00	0.87	0.00	0.48	0.00	1.00
**cis-Myrtanol**	0.00	0.00	0.00	0.00	0.00	0.00	0.14
**Dihydrocarvyl acetate**	0.00	5.58	0.00	0.00	0.00	0.00	0.00
**(-)-cis-Isopiperitenol**	0.00	0.00	0.00	0.32	0.00	0.00	0.00
**cis-Verbenyl acetate**	0.00	19.80	0.00	34.78	0.00	0.00	0.00
**Neoisoisopulegol**	0.00	0.12	0.00	0.00	0.00	0.17	0.18
**Rosifoliol**	0.00	0.00	0.44	0.00	0.00	0.00	0.00
**2-Naphthalenemethanol, decahydro-alpha,alpha,4a-trimethyl-8-methylene-, [2R-(2alpha,4aalpha,8abeta)]-**	0.00	0.00	0.24	0.00	0.00	0.00	0.00
**beta Bourbonene**	0.00	0.00	0.00	0.00	0.00	0.69	0.00
**Espatulenol**	0.00	0.13	0.00	0.00	0.00	0.26	0.00
**(+)-gamma-cadinene**	0.00	0.16	0.00	0.00	0.00	0.00	0.00
**(-)-Guaia-6,9-diene**	0.00	0.00	0.00	0.00	0.00	0.00	0.10
**Humulene epoxide II**	0.00	0.00	0.00	0.00	0.00	0.00	0.16
**.beta.-Copaene**	0.00	0.00	0.00	0.00	0.42	0.00	0.00
**(1aR,4S,4aR,7R,7aS,7bS)-1,1,4,7-Tetramethyldecahydro-1H-cyclopropa[e]azulen-4-ol**	0.00	0.00	0.75	0.00	0.00	0.00	0.00
**Selina-5,11-diene**	0.00	0.00	0.18	0.00	0.00	0.00	0.00
**Bicyclogermacrene**	3.52	3.70	0.00	0.00	0.00	1.78	0.00
**Cubebol**	0.00	0.22	0.00	0.00	0.83	0.00	0.00
**(-)-alpha-Isocomene**	1.67	0.00	0.00	0.00	0.00	0.00	0.00
**alpha-Cadinol**	0.54	0.15	0.00	0.00	0.00	0.10	0.00
**alpha-Cubebene**	0.00	0.00	0.13	0.00	0.30	0.15	0.00
**gamma-Muurolene**	0.00	0.00	0.00	0.00	0.73	0.00	0.00
**Cedrelanol**	0.00	0.32	0.00	0.00	0.14	0.00	0.00
**1H-Cycloprop[e]azulen-4-ol, decahydro-1,1,4,7-tetramethyl-, [1aR-(1a.alpha.,4.beta.,4a.beta.,7.alpha.,7a.beta.,7b.alpha.)]-**	0.00	0.00	0.27	0.00	0.00	0.00	0.00
**(-)-alpha-Gurjunene / 4-Aromadendrene**	0.00	0.00	2.15	0.00	0.43	0.11	0.00
**1H-Cycloprop[e]azulene, 1a,2,3,5,6,7,7a,7b-octahydro-1,1,4,7-tetramethyl-, [1aR-(1a.alpha.,7.alpha.,7a.beta.,7b.alpha.)]-**	0.00	0.00	1.46	0.00	0.00	0.00	0.00
**.beta.-Maaliene**	0.00	0.00	0.10	0.00	0.00	0.00	0.00
**Calarene**	0.00	0.00	0.33	0.00	0.00	0.00	0.00

**1H-Cycloprop[e]azulen-7-ol, decahydro-1,1,7-trimethyl-4-methylene-, [1ar-(1a.alpha.,4a.alpha.,7.beta.,7a.beta.,7b.alpha.)]-**	2.24	2.99	0.00	0.00	1.08	1.61	0.00
**(-)-beta-Cubebene**	0.00	0.00	0.00	0.00	0.00	0.48	0.00
**Acorenone B**	0.00	0.11	0.00	0.00	0.00	0.00	0.00
**alpha-Curcumene**	0.00	4.78	0.00	0.00	0.00	0.00	0.00
**alpha-Zingiberene**	0.00	3.23	0.00	0.00	0.00	0.00	0.00
**Alloaromadendrene**	0.00	0.00	0.00	0.00	0.00	0.59	0.00
**Aromandendrene**	0.00	0.00	6.27	0.00	0.55	0.00	0.00
**gamma-Gurjunene**	0.00	0.00	1.68	0.00	0.00	0.22	0.00
**Azulene, 1,2,3,5,6,7,8,8a-octahydro-1,4-dimethyl-7-(1-methylethenyl)-, [1S-(1.alpha.,7.alpha.,8a.beta.)]-**	0.00	0.00	0.00	0.00	0.00	0.29	0.00
**beta-Cedrene**	0.00	0.19	0.00	0.00	0.00	0.00	0.00
**beta-Curcumene**	0.00	2.72	0.00	0.00	0.00	0.00	0.00
**beta-Copaene**	0.62	0.00	0.00	0.00	0.00	0.00	0.00
**(+)-Chrysanthenone**	0.00	0.00	0.00	0.00	0.00	0.00	0.39
**alpha-Bergamotene**	0.00	0.00	0.00	0.28	0.00	0.00	0.00
**Bicyclosesquiphellandrene**	0.00	0.00	0.00	0.00	0.52	0.00	0.00
**cis-.beta.-Farnesene**	0.00	0.00	0.00	0.16	0.00	0.00	0.00
**cis-beta-Elemeno**	0.00	0.32	0.00	0.00	1.02	0.00	0.00
**Cadina-1(10),4-diene**	1.66	0.00	0.00	0.00	0.00	0.00	0.00
**Cyperene**	2.14	0.00	0.00	0.00	0.00	0.00	0.00
**Isoaromadendrene epoxide**	0.00	0.12	0.00	0.00	0.00	0.00	0.00
**Isospathulenol**	0.00	0.47	0.00	0.00	0.00	0.00	0.00
**Isoledene**	0.00	0.00	0.35	0.00	0.15	0.00	0.00
**Humulene**	0.61	0.00	0.00	0.00	3.08	0.00	7.53
**Naphthalene, 1,2,3,5,6,8a-hexahydro-4,7-dimethyl-1-(1-methylethyl)-, (1S-cis)-**	0.00	0.60	0.23	0.19	2.30	2.84	0.13
**delta-Selinene**	0.00	0.00	0.16	0.00	0.00	0.00	0.00
**Naphthalene, 1,2,3,4,4a,7-hexahydro-1,6-dimethyl-4-(1-methylethyl)-**	0.00	0.00	0.00	0.00	0.17	0.00	0.00
**Beta-Cadinene**	0.00	0.00	0.00	0.00	0.00	0.00	0.35
**Nerolidol**	0.87	1.96	0.00	0.00	0.32	0.00	0.00
**Silphinene**	0.47	0.00	0.00	0.00	0.00	0.00	0.00
**Selin-6-en-4.alpha.-ol**	0.00	0.00	0.00	0.52	0.00	0.00	0.00
**.beta.-Copaene**	0.00	0.00	0.00	0.00	0.14	0.00	0.00
**Ylangene**	0.00	0.00	0.00	0.00	0.24	0.00	0.56
**alpha-Farnesene**	6.73	0.00	0.00	0.00	0.00	0.31	0.28
**alpha-Muurolene**	0.00	0.00	0.00	0.00	0.62	0.00	0.30
**beta-Isocomene**	1.78	0.00	0.00	0.00	0.00	0.00	0.00
**beta-Bisabolene**	2.99	0.00	0.00	0.19	0.00	0.00	0.00
**beta-Pinene**	0.91	0.00	8.21	0.00	3.37	0.00	0.00
**Germacrene D**	18.99	2.31	0.00	0.00	13.10	4.57	0.00

**Germacrene D-4-ol**	0.38	0.72	0.00	0.00	0.78	0.17	0.00
**Globulol**	0.00	0.00	3.77	0.00	0.00	0.14	0.00
**3-Methyl-2-(2-methyl-2-butenyl)-furan**	0.00	0.18	0.00	0.00	0.00	0.00	0.00

Table S2. Taxonomic data, extraction yield, and physical characteristics (color) of the essential oils analyzed

Species	Scientific name	Essential oil yield (V/P%)	Essential oil color
Anis	***Tagetes filifolia***	0.47	Pale yellow to colorless
Cedron	***Aloysia citrodora***	0.09	Yellow
Eucalipto	***Eucalyptus globulus***	0.99	Pale yellow to colorless
Hierba luisa	***Cymbopogon citratus***	0.92	
Matico	***Piper aduncum***	0.88	
Manzanilla	***chamaemelum nobile***	0.04	Intense yellow
Poleo	***Minthostachys mollis***	0.44	Pale yellow to colorless
Romero	***Rosmarinus officinalis***	0.42	
